# Pan-metagenome reveals the abiotic stress resistome of cigar tobacco phyllosphere microbiome

**DOI:** 10.3389/fpls.2023.1248476

**Published:** 2023-12-21

**Authors:** Zhenhua Wang, Deyuan Peng, Changwu Fu, Xianxue Luo, Shijie Guo, Liangzhi Li, Huaqun Yin

**Affiliations:** ^1^ Zhangjiajie Tobacco Company of Hunan Province, Zhangjiajie, China; ^2^ School of Minerals Processing and Bioengineering, Central South University, Changsha, China; ^3^ Key Laboratory of Biometallurgy of Ministry of Education, Central South University, Changsha, China

**Keywords:** phyllosphere, abiotic stress resistance, metagenome, virus, auxiliary metabolic gene

## Abstract

The important role of microbial associations in mediating plant protection and responses to abiotic stresses has been widely recognized. However, there have been limited studies on the functional profile of the phyllosphere microbiota from tobacco (*Nicotiana tabacum*), hindering our understanding of the mechanisms underlying stress resilience in this representative and easy-to-cultivate model species from the solanaceous family. To address this knowledge gap, our study employed shotgun metagenomic sequencing for the first time to analyze the genetic catalog and identify putative plant growth promoting bacteria (PGPB) candidates that confer abiotic stress resilience throughout the growth period of cigar tobacco in the phyllosphere. We identified abundant genes from specific bacterial lineages, particularly *Pseudomonas*, within the cigar tobacco phyllospheric microbiome. These genes were found to confer resilience against a wide range of stressors, including osmotic and drought stress, heavy metal toxicity, temperature perturbation, organic pollutants, oxidative stress, and UV light damage. In addition, we conducted a virome mining analysis on the metagenome to explore the potential roles of viruses in driving microbial adaptation to environmental stresses. Our results identified a total of 3,320 scaffolds predicted to be viral from the cigar tobacco phyllosphere metagenome, with various phages infecting *Pseudomonas, Burkholderia, Enterobacteria, Ralstonia*, and related viruses. Within the virome, we also annotated genes associated with abiotic stress resilience, such as alkaline phosphatase D (*phoD*) for nutrient solubilization and glutamate-5-semialdehyde dehydrogenase (*proA*) for osmolyte synthesis. These findings shed light on the unexplored roles of viruses in facilitating and transferring abiotic stress resilience in the phyllospheric microbiome through beneficial interactions with their hosts. The findings from this study have important implications for agricultural practices, as they offer potential strategies for harnessing the capabilities of the phyllosphere microbiome to enhance stress tolerance in crop plants.

## Introduction

The phyllosphere, also known as the phylloplane, refers to the aerial foliage surface where microbes thrive. It serves as a protective barrier against various biotic and abiotic stresses, including temperature changes, ultraviolet (UV) radiation, drying out, and nutrient deficiency (Zhang et al., 2022). Traditionally, the phyllosphere was thought to be inhospitable to microbes, but subsequent research has shown that it harbors a diverse array of microbial taxa that have adapted to these challenging conditions (Vorholt, 2012). Microbial communities in the phyllosphere rely on specific resilience mechanisms to withstand external stresses. These mechanisms incorporate various strategies, such as pigment production to protect against intense UV radiation, the secretion of extracellular polysaccharides (EPS) or biosurfactants to facilitate surface attachment and prevent desiccation, and the production of chemical compounds to compete for resources (Heredia-Ponce et al., 2021; Bashir et al., 2022). The assembly of phyllosphere microbiota is influenced by a combination of intrinsic factors, like plant genotype, age, and species, as well as biotic and abiotic environmental factors, including climate, geographical location, and properties (Shakir et al., 2021). Among the phyllosphere-associated ecosystem, bacterial species are the most abundant members, with an estimated density of 10^6^ to 10^7^ bacterial cells per square centimeter. These bacteria can play beneficial, pathogenic, or antagonistic roles in the phyllosphere (Lindow and Brandl, 2003).

The phyllospheric microbiota engages in complex, dynamic, and multipartite interactions that significantly contribute to plant health and productivity (Lindow and Brandl, 2003). These interactions between plants and associated microbial communities are important drivers of terrestrial ecosystems (van der Putten et al., 2013). Moreover, the phyllosphere microbiota exhibits diverse functional roles, including one-carbon conversion (Knief et al., 2012), fixation of nitrogen, nitrification (Fürnkranz et al., 2008), modulation of plant metal transporters (Zhou et al., 2020) and bioremediation of aerial hydrocarbon pollutant (Ali et al., 2012; Franzetti et al., 2020).

Microbial colonization on plant surfaces has also been shown to promote plant growth through various mechanisms. These include increased antioxidant defense enzyme activity (Mastouri et al., 2012), production of volatile organic compounds (VOCs)/phytohormones to regulate plant communication and development (Taghavi et al., 2009; Liu and Zhang, 2015), protection against foliar pathogens (Innerebner et al., 2011), decomposition of toxic substances (Vorholt, 2012) and enhancement of stress tolerance (Márquez et al., 2007; Patel et al., 2017). For instance, rice seedlings inoculated with specific phyllosphere bacterial strains have demonstrated improved survival under drought stress, along with enhanced nutrient availability, exopolysaccharide levels, phytohormones, soluble sugars, chlorophyll, and total protein (Arun et al., 2020). Similarly, inoculation with the rice phyllosphere bacteria *Bacillus megaterium* strain PB50 has been found to enhance the drought tolerance of *Oryza sativa* (rice) pots (Devarajan et al., 2021). These beneficial effects on plant growth have been reported in various other studies as well (Enya et al., 2007; Batool et al., 2016; Fu et al., 2016).

While the beneficial properties of phyllosphere microbes are known, much remains to be explored regarding their genetic repertoire (Meyer and Leveau, 2012). Additionally, the mutualistic aspects of plant-microbe interactions, such as stress tolerance and plant defense, in the phyllosphere require further in-depth study.

In addition to bacteria, abundant viruses, including bacteriophages (phages), have been discovered on the phyllosphere. These viruses can infect and replicate within bacteria that reside on the phyllosphere, impacting the composition and diversity of the associated bacterial communities. Phages can also exert selective pressure on bacterial populations, leading to the elimination or reduction of specific bacterial populations (Forero-Junco et al., 2022). Furthermore, the prevalence of certain phages can vary across environments and plant species, influencing the composition of associated bacterial communities. Interestingly, beneficial effects of viral infections in host plants have been documented, as certain plant virus strains enhance the abiotic stress resistance of their hosts. For example, cucumber mosaic virus (CMV) strain Fny, bromo mosaic virus (BMV) strain Russian, tobacco mosaic virus (TMV) U1 strain, and tobacco rattle virus (TRV) have been found to enhance the heat, cold, or drought resistance of their plant hosts (Xu P. et al., 2008; Roossinck, 2013; Westwood et al., 2013). Thus, the role of viruses in the phyllosphere and their impact on plant-microbe interactions warrant further investigation.

Tobacco (*Nicotiana tabacum*) is a leafy, annually-grown solanaceous crop of significant economic importance, cultivated worldwide for thousands of years. China is one of the major tobacco producers, accounting for 39.06% of global tobacco production (http://www.fao.org/faostat/en/#data/QC). This highlights the agricultural significance of tobacco and its role in the global market. Moreover, tobacco with broad environmental adaptability serves as a valuable model plant for studying various physiological processes and plant-pathogen interactions. Researchers often turn to tobacco as a model due to its well-established experimental systems and the ease of manipulation in laboratory settings (Dai et al., 2022). Tobacco is typically grown during the summer and harvested at the end of August. Being exposed to excessive solar/ultraviolet radiation, diurnal temperature fluctuations, and occasional heavy rainfall during growth, tobacco leaves offer a unique opportunity to investigate microbial communities under strong abiotic stresses. Consequently, the phyllosphere of solanaceous crops, such as tobacco, serves as a suitable model system for investigating the dynamics of microbial populations and their interactions in the face of environmental challenges. This is due to the significant environmental heterogeneity and intricate ecological interactions that occur on the surfaces of leaves (Meyer and Leveau, 2012; Xing et al., 2022).

Methods for studying the structure and biodiversity of the plant phyllospheric microbiome have evolved significantly in recent years. These methods enable researchers to gain insights into the complex microbial communities that inhabit the phyllosphere of solanaceous crops like tobacco. Early studies were limited to culture-dependent methods, but the introduction of denaturing gradient gel electrophoresis (DGGE) by Yang et al. revolutionized the field (Yang et al., 2001). However, traditional culture methods are time-consuming and have low throughput, often leading to an underestimation of microbial population sizes and biodiversity (Dai et al., 2022). Recent advancements in low-cost high-throughput (Knight et al., 2018) and next-generation sequencing technologies (Soucy et al., 2015) have overcome these limitations, enabling researchers to explore microbial communities in greater detail and with higher resolution.

High-throughput sequencing methods, such as targeted sequencing of phylogenetic markers like 16S rRNA for bacteria and ITS for fungi, have been successfully applied in the study of the tobacco foliage microbiome (Chen et al., 2020; Huang et al., 2021; Zheng et al., 2022). While in-silico predictions based on phylogenetic marker genes like the 16S rRNA gene can provide valuable insights into microbial diversity and community composition, there are several limitations such as the lack of functional information, limited resolution and bias towards abundant taxa (Pan et al., 2023).

More advanced metagenomic and metaproteomic shotgun sequencing approaches have allowed for faster and more accurate characterization of taxonomic and functional profiles of microbiomes at the species level, encompassing multiple domains such as bacteria and fungi. These approaches have been applied to various plant phyllospheric microbiomes, including those of sugarcane (Khoiri et al., 2021), brick tea (Wang et al., 2021) and neotropical forest (Lajoie et al., 2020).

However, metagenomic studies of the tobacco phyllospheric microbiome are limited, and functional characterization is primarily based on in-silico predictions using marker genes like the 16S rRNA gene. This limitation hinders our understanding of microbial functions and their adaptation to the tobacco phyllosphere, as well as the factors influencing microbiome dynamics over time and space. Thus, for enhancing plant health and growth and manage disease outbreaks, there is a need for further research to gain a more accurate and in-depth understanding of the tobacco microbial community and its functional repertoire, particularly in relation to abiotic stress responses.

In this study, we conducted pan-metagenomic investigations of the phyllosphere (leaf-epiphytic) microbiome of cigar tobacco from Hunan province, China. This region has a long history of tobacco production and is also affected by bacterial wildfire disease. To capture temporal dynamics, our investigations were performed throughout the tobacco growth season. Using genome assembly and annotation, we characterized the taxonomic and functional profiles of the cigar tobacco phyllospheric microbiome. Specifically, we focused on the “resistome,” which encompasses the complete set of genes or genetic elements involved in conferring resistance to various abiotic stresses, such as temperature, drought, salinity, and chemical pollutants. This pan-metagenomic approach allowed us to comprehensively analyze the cigar tobacco phyllospheric microbiome and gain valuable insights into its functional potential in relation to stress resistance.

Furthermore, our study also identified viral sequences within the metagenome scaffolds. This finding highlights the role of viruses as horizontal gene transfer (HGT) agents in facilitating the transfer of metabolic and stress resistance genes among the phyllospheric microbiota. HGT refers to the transfer of genes between organisms that do not have a direct parent-offspring relationship, contrasting with vertical gene transfer that occurs through reproduction. In the context of microbial communities, HGT enables the exchange of genetic material, including genes or DNA fragments, between different microorganisms (Huang, 2013; Daubin and Szöllősi, 2016).

## Results and discussion

### Pan-metagenome analyses and taxonomic composition

A total of 444,193 protein-coding genes were annotated from all three groups of metagenomes representing phyllosphere (leaf-epiphytic) microbiota from cigar tobacco. This diversity greatly exceeds the previously reported 4,587 metagenomic orthologous genes from tropical tree phyllosphere communities (Lajoie et al., 2020), emphasizing the exceptional richness and genetic potential of the cigar tobacco phyllospheric microbiota. It suggests that the phyllospheric microbiota associated with cigar tobacco harbors a vast repertoire of genetic elements, which likely contributes to its ability to adapt, interact with the host plant, and engage in various functional processes. This extensive genetic diversity in the metagenomes is indicative of the vast microbial species richness and functional capacity present in the phyllosphere of cigar tobacco.

**Figure 1:**
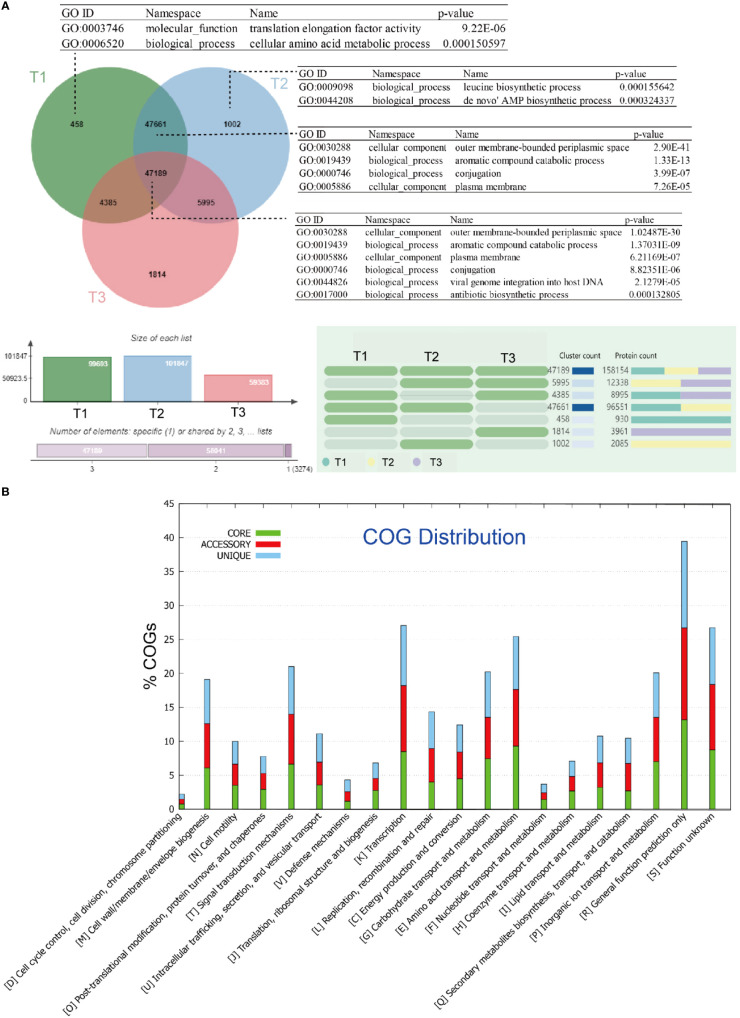
Clustering and comparative metagenomic analyses of three group of samples: **(A)** Venn diagram showing the number of genes shared by all strains (i.e., the core genome), the number of genes shared by partial strains (i.e., the accessory genome), and the number of strain-specific genes (i.e., the unique gene) in the tested strains. The gene ontology (GO) categories were determined using OrthoVenn, and the hypergeometric test with a *p*-value < 0.05 was applied to find enriched GO in the clusters; **(B)** Bar chart showing functional proportions (based on COG categories) of different parts of the pan-metagenome (i.e., core, accessory, unique).

Among these genes, 47,189 gene families were found to be shared by all groups of metagenome samples in this study. These gene families are significantly enriched in gene ontology (GO) terms such as aromatic compound catabolic process (GO:0019439), conjugation (GO:0000746), viral genome integration into host DNA (GO:0044826), and antibiotic biosynthetic process (GO:0017000) (**Figure 1A**). The number of unique gene families shows an increasing trend from group I (samples collected in June; 458), to group II (samples collected in July; 1,002), and group III (samples collected in August; 1,814), indicating an increasing diversity of metagenomic genes over time.

In our study, the core gene set primarily consists of gene families that are annotated as clusters of orthologous groups (COG) categories, including post-translational modification [O], translation, ribosomal structure, and biogenesis [J], energy production and conversion [C], carbohydrate transport and metabolism [G], amino acid transport and metabolism [E], and nucleotide transport and metabolism [F] (**Figure 1B**).

The enrichment of these COG categories suggests important functional roles within the core gene set. Post-translational modification [O] may be involved in protein folding, stability, and enzymatic activity regulation, which could impact the adaptation and survival of the phyllospheric microbiota on plant surfaces. Translation, ribosomal structure, and biogenesis [J] are essential for protein synthesis, suggesting that the core gene set is enriched in genes involved in protein production and regulation.

Energy production and conversion [C] indicate the presence of genes related to energy metabolism, suggesting that the core gene set includes functional traits associated with energy utilization and adaptation in the phyllosphere. Carbohydrate transport and metabolism [G] and amino acid transport and metabolism [E] categories imply that the core gene set contributes to the utilization of carbon and nitrogen sources available on plant surfaces. Additionally, nucleotide transport and metabolism [F] imply that the core gene set may possess genetic capabilities related to DNA and RNA metabolism.

Overall, the enrichment of these specific COG categories in the core gene set suggests that the phyllospheric microbiota has developed functional capabilities to interact with the plant environment, including protein regulation, energy metabolism, resource utilization, and genetic processes. These functional implications are crucial for microbial adaptation and survival on leaf surfaces.

The Kyoto Encyclopedia of Genes and Genomes (KEGG) annotation further reveals enrichment of categories related to the metabolism of cofactors and vitamins in the core gene set (see Figure S1 at https://doi.org/10.6084/m9.figshare.21257361.v1). On the other hand, enriched GO terms in the unique gene families of group I include translation elongation factor activity (GO:0003746), and correspondingly, in group II include leucine biosynthetic process (GO:0009098), *de novo*’ AMP biosynthetic process (GO:0044208); while in the shared gene families of group I and group II, aromatic compound catabolic process (GO:0019439), conjugation (GO:0000746), are again significantly enriched.

Model extrapolation revealed an ‘‘open’’ pan-metagenome fitted into a power-law regression function [*Ps* (n) = 57974.6n^0.4196^] with a calculated exponent γ falling in the range between 0 and 1, while the core metagenome was fitted into an exponential regression [*Fc* (n) = 86462.6e ^-0.414371n^]. This indicates that our sampling of microbial taxa from cigar tobacco phyllosphere is still unsaturated.

In the cigar tobacco phyllosphere, bacteria were found to be the most abundant microbial colonizers, comprising approximately 99.9% of the total community, followed by viral communities at around 0.04%. Among the bacterial taxa, the phylum Proteobacteria dominated, accounting for about 98.1% of the community, with the phylum Firmicutes representing a minor proportion of around 0.05%.

Within Proteobacteria, the class Gammaproteobacteria was the most abundant, representing approximately 91% of the community, followed by the class Alphaproteobacteria at around 8%. Among the orders of Gammaproteobacteria, Enterobacterales and Pseudomonadales were the dominant groups, making up around 58% and 41% of the Gammaproteobacteria community, respectively.

Interestingly, the study revealed variations in the abundance of Enterobacterales and the wildfire disease pathogen *Pseudomonas syringae* among different sampling time points (T1, T2, and T3). The T3 group, corresponding to late August, exhibited a higher abundance of Enterobacterales (~76%) and *Pseudomonas syringae* (~2%) compared to the T1 group (~53% and ~0.07%) and T2 group (~54% and ~0.10%) (see Figure S2 at https://doi.org/10.6084/m9.figshare.21257361.v1 and Supplemented data at https://doi.org/10.6084/m9.figshare.21257256.v1).

The taxonomic analysis of the cigar tobacco phyllospheric microbiome revealed a dominance of bacteria, particularly those belonging to the Proteobacteria phylum. Among these, the bacterial wildfire disease pathogen *P. syringae* were found to be highly abundant, especially during late August (T3 group). This observation aligns with previous studies based on 16S rRNA amplicon analysis, which indicated a correlation between the abundance of these bacteria and the development of bacterial wildfire disease in tobacco (Wang et al., 2022).

These findings shed light on the potential dynamics and composition of the microbial community in the phyllosphere and their potential roles in cigar tobacco health and disease development. They further support the study’s goal of understanding microbial interactions and their ecological significance in the phyllosphere environment.

Furthermore, the differential abundance of specific bacterial groups and pathogens at different sampling time points may provide valuable insights into the identification of microbial signatures associated with disease progression. This information can aid in the development of effective management strategies to improve cigar tobacco health and mitigate the impact of diseases.

### Abiotic stress resistome in cigar tobacco phyllosphere

#### Osmotic and drought stress resilience

Osmotic and drought stress impose significant pressure on the nutrient uptake and cellular physiology of both plant-associated microbes and their hosts (Bashir et al., 2014; Hanin et al., 2016). To combat osmotic stress, microbes and plants employ three major mechanisms: maintaining cellular ion homeostasis, enhancing cell barriers, and utilizing compatible solute protectants such as trehalose, proline, betaine, and sarcosine (Behr et al., 2015). Plant-microbial interactions play a crucial role in osmotic adjustment, as they facilitate the secretion of metabolites that help maintain osmotic balance (Shaffique et al., 2022). Exopolysaccharides (EPSs) are hydrophilic macromolecules composed of long-chain polymers with repeating sugar units (Ilyas et al., 2020; Nadeem et al., 2021), and they form protective biofilms that enhance water retention in the microbe-sheath and regulate the distribution of carbon sources to mitigate the effects of aridity and dehydration caused by abiotic stress (Xu J. et al., 2008; Naseem et al., 2018).

**Figure 2:**
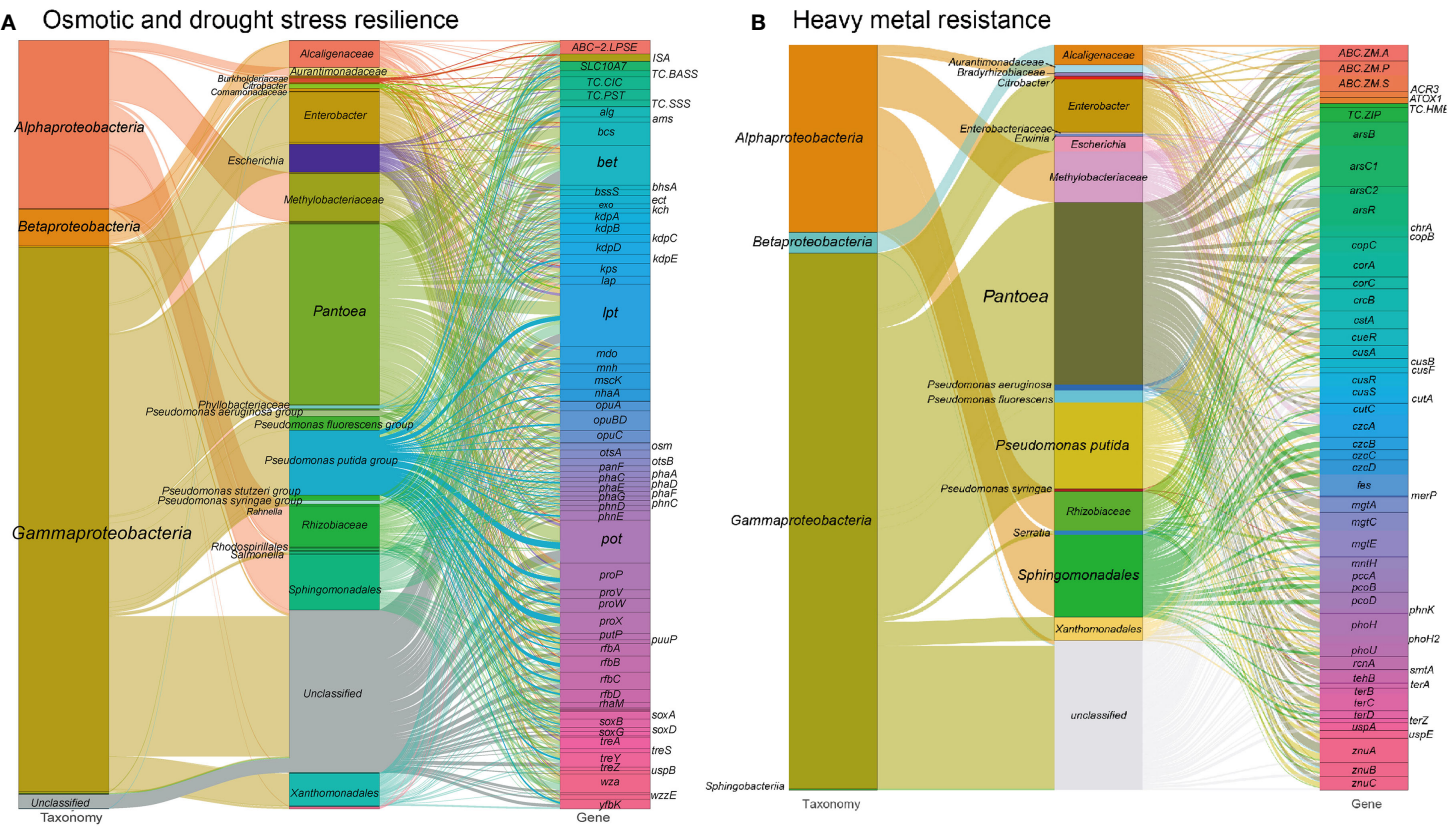
Sankey diagram showing the taxonomic and functional profiles of genes conferring abiotic stress resilience in the cigar tobacco phyllospheric microbiome: **(A)** Osmotic and drought stress resilience; **(B)** Heavy metal resistance. Details for gene abbreviations can be found in Table S1 at https://doi.org/10.6084/m9.figshare.21257352.v1.

In our analysis, we identified gene orthogroups related to osmotic stress resistance in the metagenome of the tested tobacco phyllosphere. Details of these gene orthogroups can be found in Table S1 at https://doi.org/10.6084/m9.figshare.21257352.v1 and are illustrated in **Figure 2A**. These genes play crucial roles in various mechanisms that contribute to osmotic stress resistance:

- Universal stress regulators, such as osmotically inducible lipoprotein (*osmBE*), universal stress protein (*uspABCEG*), biofilm regulator (*bssRS*), biofilm protein (*tabA*), help microorganisms adapt to osmotic stress by coordinating cellular responses.
- Ion transporters, including voltage-gated potassium channel (*trkA*), potassium-dependent mechanosensitive channel (*kefABCFG*), potassium-transporting ATPase (*kdpABCDE*), multicomponent K^+^:H^+^ antiporter (*phaACDEFG*), multicomponent Na^+^:H^+^ antiporter (*nhaAB*, *mnhABCDEG*), sodium channel (VGSC), sodium/proline symporter (*putP*), sodium/bile acid symporter (SLC10A7, TC.BASS), solute:Na^+^ symporter (TC.SSS) and chloride channel (*yfbK*, TC.CIC), play a vital role in maintaining cellular homeostasis under osmotic stress.
- Biosynthesis/export proteins involved in enhancing the cell barrier, including the production of lipopolysaccharide (LPS) precursor rhamnose (dTDP-l-rhamnose synthase: *rfbABCD* (Parakkottil et al., 2010), L-rhamnose mutarotase and transporter (*rhaMPQST*), cellulose synthase (*bcsBC*), and capsular polysaccharide or lipopolysaccharide biosynthesis/export protein (*kpsTS, etc.*);
- Biosynthesis and transport proteins involved in the synthesis of protectant sugars, such as ectoine (*ectABCD*), succinoglycan (*exoFHIQXY*), glycine betaine (*betABCLT, BHMT*, *CMO*), amylovoran (*amsFL*), alginate (*algEFIJKX*), trehalose (*treASXYZ, otsAB*), glucans (*mdoCG*), sarcosine (*soxABDG*) as well as osmoprotectant transport system (*opuABDC*), and glycine proline/betaine transporter (*proPVWX*). These sugars act as osmoprotectants, helping to maintain cellular integrity and functionality.

These osmotic stress resistance genes are primarily encoded by specific bacterial groups in the metagenome. Gammaproteobacteria, such as *Enterobacter* (6.5%), *Pseudomonas* (13.3%), *Pantoea* (23.4%), and Alphaproteobacteria including *Rhizobiaceae* (5.3%), *Methylobacteriaceae* (6.2%), and *Sphingomonadales* (7.2%), play a significant role in encoding these proteins. Specially, *Sphingomonadales* (16.0%) and *Pantoea* (20.5%) are the primary contributors to the encoding of lipopolysaccharide biosynthesis/export proteins. *Methylobacteriaceae* (11.8%) and *Pantoea* (18.5%) are predominantly involved in encoding ion transporters. Besides, *Pseudomonas* (14.3%), *Pantoea* (18.2%) and *Sphingomonadales* (9.3%) are the major contributors to the synthesis of compatible solutes. Among these taxa, *Enterobacter* may contribute to nutrient cycling by participating in the breakdown of organic matter and nutrient solubilization, indirectly benefiting the plants. Most *Pseudomonas* species produce plant growth-promoting substances, including IAA, siderophores, and volatile organic compounds, which can enhance plant growth and trigger defense mechanisms against pathogens. *Pantoea* spp. have been reported as nitrogen-fixing bacteria, having the ability to convert atmospheric nitrogen into plant-usable forms (Singh et al., 2020).

Microorganisms indirectly mitigate osmotic pressure in plant cells by accelerating trehalose biosynthesis, maintaining osmolyte concentrations, and stabilizing turgor pressure (Kahraman et al., 2019; Shaffique et al., 2022). The application of osmotic-stress-resilient microbiota to plants can induce the secretion of organic acids and mineral solubilization, thereby increasing nutrient availability, metabolic rate, and sustaining osmoregulation in plant cells (Chen and Jiang, 2010; Shaffique et al., 2022). Plant-growth-promoting bacteria (PGPB) contribute to osmotic adjustment by generating a low water potential gradient in the cytosol, maintaining turgor pressure, osmotic adjustment, and improving stress tolerance in plant cells (Shaffique et al., 2022).

Furthermore, compatible solutes produced by the microbiome protect both the microbiome itself and the plant host against drought, heat, or cold stress (Bashir et al., 2014; Hanin et al., 2016). The biosynthesis of osmoprotectants and the production of EPS enriched in the tested metagenome may reflect adaptations of microbial inhabitants by enhancing attachment to surfaces and offering resistance to environmental pressures and plant defenses (Rastogi et al., 2013). In a recent study by de Sousa et al. (de Sousa et al., 2022), it was demonstrated that Pseudomonas spp. rely on increased synthesis of exopolysaccharides (EPSs) to cope with osmotic stress and protect cells from desiccation in the phyllosphere. This finding supports our previous study, where we identified an enrichment of *Pseudomonas* spp. (LDA = 5.29) during the T3 period, which coincided with the predicted enrichment of genes associated with osmoprotectant biosynthesis (Wang et al., 2022). This suggests a correlation between the abundance of Pseudomonas spp. and the presence of osmoprotectant biosynthesis genes, particularly during periods of intense sun exposure and drought stress such as the T3 period.

Similarly, the increased production of EPSs under osmotic stress was reported in rhizobacteria *Pseudomonas aeruginosa* and *Bacillus endophyticus* (Ghosh et al., 2019). Finally, the accumulation of compatible solutes (glycine-betaine and ectoine) in the biocontrol agent *Pantoea* spp. serves as an osmotic stress adaptation (Teixidó et al., 2005).

These findings have practical implications and can contribute to various applications. For example, the knowledge gained from studying these osmotic stress-related proteins could lead to the development of microbial-based strategies for enhancing crop resilience. Understanding the mechanisms underlying osmotic stress resistance may also help improve plant adaptation to challenging environments. Ultimately, these advancements could reduce the reliance on traditional chemical stressors in agriculture and promote more sustainable practices.

#### Heavy metal resistance

Heavy metal contamination can have detrimental effects on plant health and the microbial communities associated with both the rhizosphere and phyllosphere. However, it is worth noting that the phyllospheric surface can act as an important reservoir for toxic element pollutants, providing significant insight into the complex interactions between plants and heavy metals (Sánchez-López et al., 2018). Furthermore, the presence of metal-resistant plant growth-promoting bacteria (PGPB) in plant spheres has been shown to enhance plant tolerance against heavy metal stress (Mishra et al., 2017; Zhou et al., 2020).

In our analysis of the metagenome from the cigar tobacco phyllosphere, a diverse range of 948 gene families were predicted to confer resistance against various heavy metals (**Figure 2B** and see Table S1 at https://doi.org/10.6084/m9.figshare.21257352.v1). These gene families mainly consist of metal ion transport proteins that facilitate the efflux of cytosolic toxic metal ions and metal reductases that convert metal ions to less toxic forms. Examples of these genes include those associated with arsenate/arsenite, chromate, copper, fluoride, nickel/cobalt, mercuric, manganese, tungstate, zinc/manganese, and tellurite resistance. Notably, the metal resistance gene orthogroups in our metagenome were predominantly encoded by *Pantoea* (~23.3%), *Pseudomonas* (~13.1%), Sphingomonadales (~10.3%), and Methylobacteriaceae (~6.5%) (Table S1). For instance, Methylobacteriaceae exclusively encoded the HME family heavy-metal exporter (TC.HME), while *Pseudomonas* predominantly encoded thioredoxin arsenate reductase (*arsC2*), ZIP family zinc transporter (TC.ZIP), zinc transport system (*znuABC*), and periplasmic copper chaperone A (*pccA*) genes. Moreover, Sphingomonadales and Methylobacteriaceae, along with *Pantoea*, played a significant role in encoding the resistance nodulation cell division (RND)-type cobalt-zinc-cadmium efflux pump (*czcABCD*) genes.

Consistent with previous studies, *Pantoea* spp. and *Pseudomonas* spp. have demonstrated potential for the bioremediation of metal-contaminated soils (Patel et al., 2016; Audu et al., 2020), while Sphingomonadales have been found inhabiting areas contaminated with high levels of metals and organic pollutants (Girardot et al., 2020). Additionally, it has been shown that the endophytic *Sphingomonas* sp. LK11 exhibits phytotoxic mitigation of Cr(VI) in soybean plants (Bilal et al., 2018).

**Figure 3:**
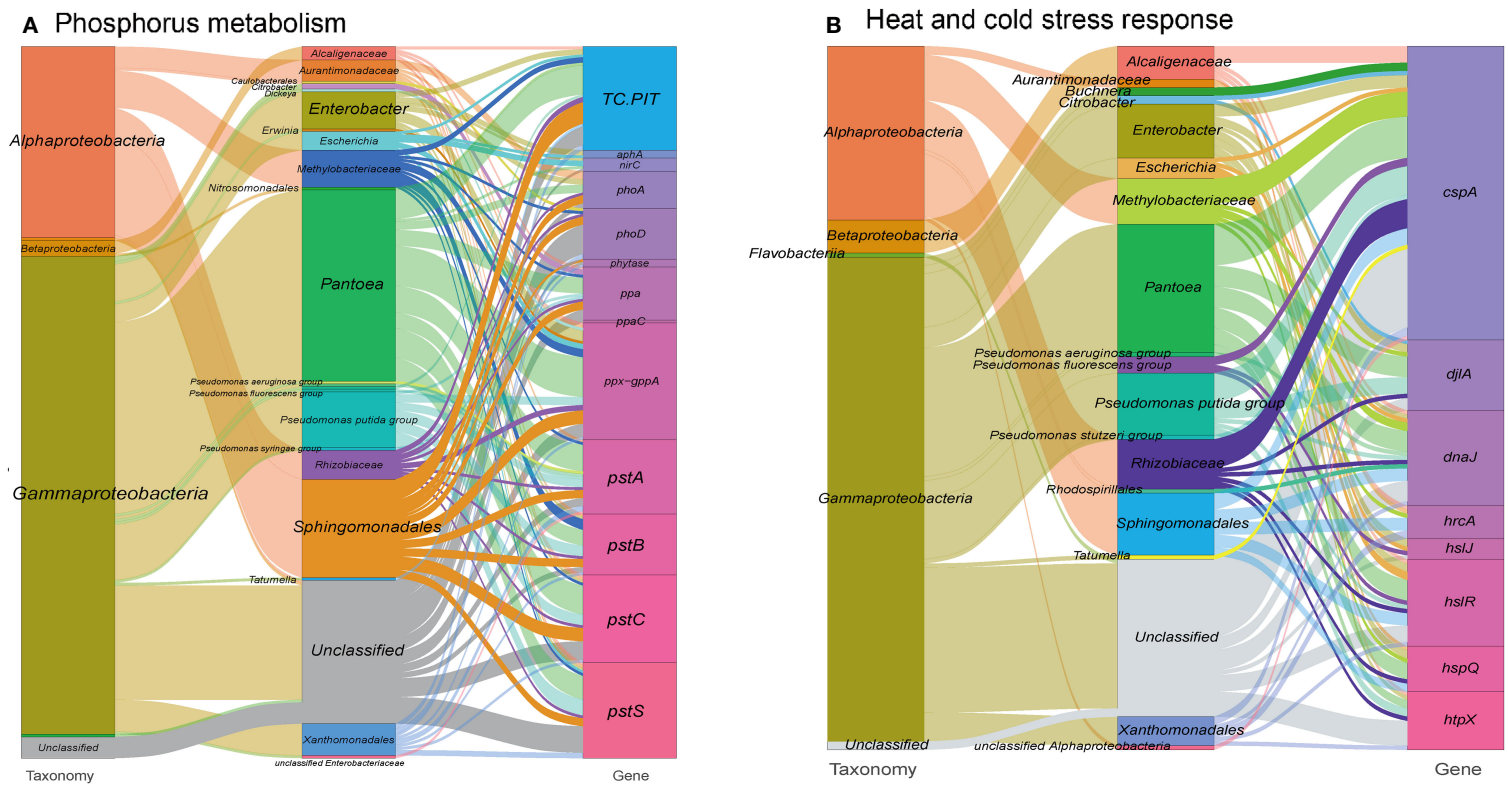
Sankey diagram showing the taxonomic and functional profiles of genes conferring abiotic stress resilience in the cigar tobacco phyllospheric microbiome: **(A)** Phosphorus metabolism; **(B)** Heat and cold stress response. Details for gene abbreviations can be found in Table S1 at https://doi.org/10.6084/m9.figshare.21257352.v1.

In addition to metal resistance genes, we also detected phosphatase and phosphorus uptake-related genes (313 gene orthogroups) in the metagenome (**Figure 3A** and see Table S1 at https://doi.org/10.6084/m9.figshare.21257352.v1). These genes may facilitate the solubilization of phosphorus, increasing its availability in the extracellular space, which is an essential nutrient for the plant host (Thapa et al., 2017). Consequently, this process might also contribute to the immobilization of toxic metal ions (Bechtaoui et al., 2021). In the other hand, a range of genes involved in biosynthesis of siderophore, which are high-affinity systems for the uptake of iron from the environment, were annotated in the metagenome. These siderophores contribute to plant nutrition and protection against phytopathogens (Scavino and Pedraza, 2013) (see Figure S3 at https://doi.org/10.6084/m9.figshare.21257361.v1 and Table S1 at https://doi.org/10.6084/m9.figshare.21257352.v1).

Overall, our findings provide comprehensive insights into the diverse repertoire of heavy metal resistance genes and their potential bacterial sources within the tobacco phyllosphere. These findings not only enhance our understanding of metal-microbe-plant interactions but also have implications for the development of strategies for phytoremediation and improving plant resilience to heavy metal stress.

### Heat and cold stress response

Large fluctuations in environmental temperature, resulting from solar radiation overexposure and changing global climate patterns, can impose significant abiotic stress on the phyllosphere of plants, leading to heat and cold stresses. To cope with these challenges, gene expansions have been observed in heat/cold shock factor gene families during adaptive evolution (Wang et al., 2018; Li et al., 2021). In our study, we identified a specific set of gene orthogroups (171 gene entries) associated with the heat/cold shock response in the metagenome of the tobacco phyllosphere (**Figure 3B** and see Table S1 at https://doi.org/10.6084/m9.figshare.21257352.v1).

Among these gene orthogroups, 71 encode cold shock proteins (cspA), primarily originating from bacterial taxa such as *Pantoea*, *Pseudomonas*, and Rhizobiaceae. Additionally, 99 gene orthogroups encode heat shock proteins, predominantly found in taxa like *Enterobacter*, Sphingomonadales, and *Pseudomonas*. These proteins function as transcription factors and molecular chaperones, working collaboratively to maintain cellular protein homeostasis (Andrási et al., 2021). For example, the heat shock protein DnaJ has been reported to protect Rubisco activity during heat stress (Wang et al., 2015). Similarly, the cold shock protein CspA promotes the proper folding of RNA molecules (Rennella et al., 2017). Furthermore, we detected 34 gene entries encoding chitinases, mainly derived from *Pseudomonas*, which have been shown to participate in cold and osmotic stress responses (Cao et al., 2019), and accordingly, we have detected 34 gene entries encoding chitinases, mainly from *Pseudomonas* (23.7%) (see Table S1 at https://doi.org/10.6084/m9.figshare.21257352.v1).

These findings demonstrate the presence of a diverse repertoire of heat and cold stress response genes in the tobacco phyllosphere metagenome, originating from various bacterial taxa. It highlights the importance of these genes in enabling plants to withstand and adapt to fluctuating temperature conditions.

### Organic pollutant resistance

Organic pollution resulting from human activities, such as the excessive use of herbicides and improper management of chemical waste, poses a significant threat to plant life. Among the various impacts of organic pollutants, phyllosphere microbes are particularly vulnerable to particulate matter, pesticides, and herbicides, which are commonly found in heavily polluted areas. For instance, a study by Chen et al. (Chen et al., 2021) investigated the effects of a broad-spectrum fungicide on bacterial communities in tobacco phyllosphere, revealing substantial differences in both core and rare taxa. Furthermore, the introduction of organic pollutant-degrading microbes to plants has been shown to mitigate the detrimental effects of organic pollutants on plants and facilitate the removal of air pollutants (Sun et al., 2015; Rajtor and Piotrowska-Seget, 2016; Franzetti et al., 2020).

**Figure 4:**
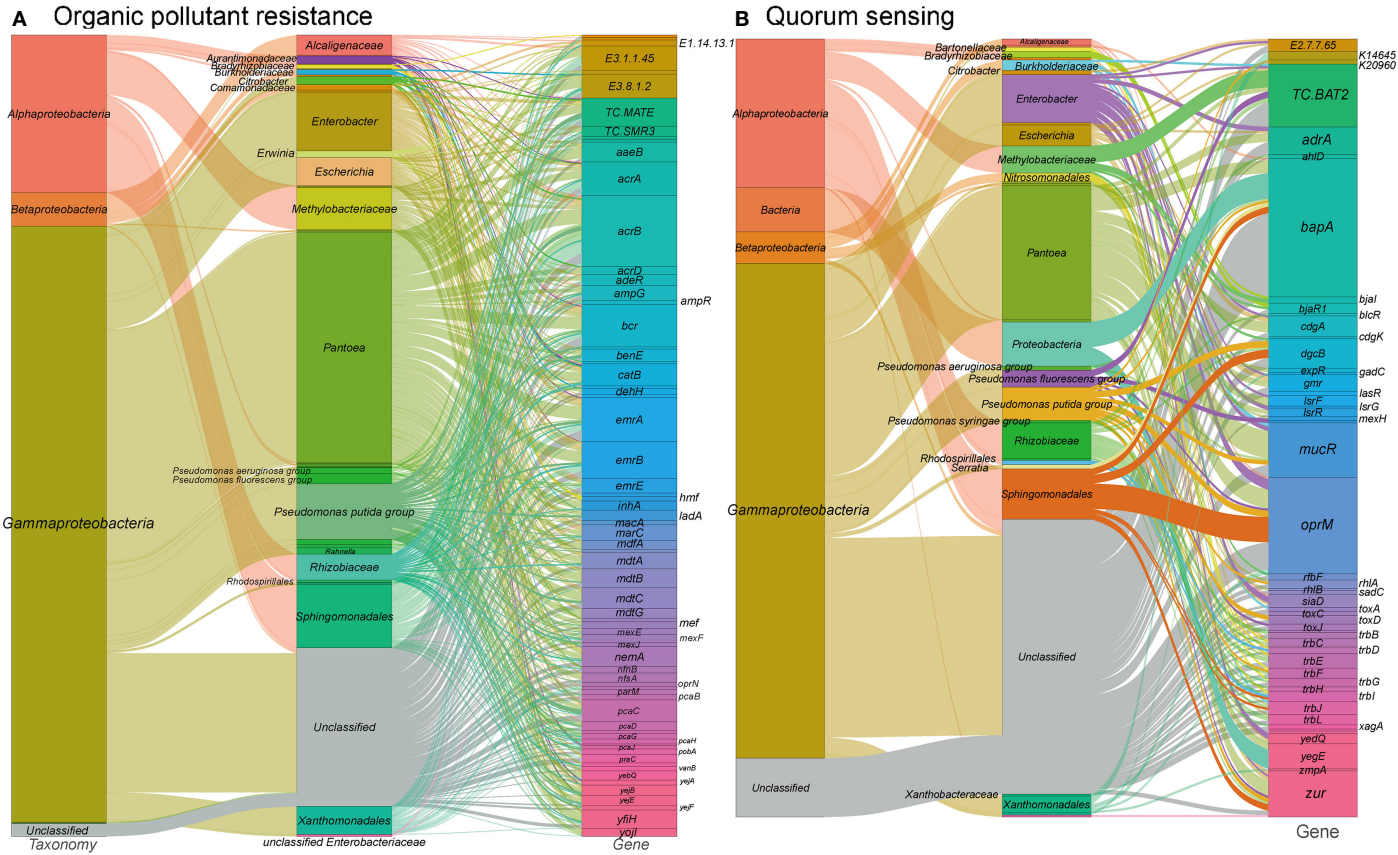
Sankey diagram depicting the taxonomic and functional profiles of genes conferring abiotic stress resilience in the metagenome of cigar tobacco phyllospheric microbiome: **(A)** Organic pollutant resistance; **(B)** Quorum sensing. Details for gene abbreviations can be found in Table S1 at https://doi.org/10.6084/m9.figshare.21257352.v1.

In our study, we identified a total of 1,309 gene families associated with xenobiotic biodegradation and metabolism (**Figure 4A** and see Table S1 at https://doi.org/10.6084/m9.figshare.21257352.v1). These gene families encompass antimicrobial resistance genes, multidrug efflux systems, beta-lactam resistance genes, as well as genes involved in the degradation of benzoate, aminobenzoate, caprolactam, chloroalkanes, chloroalkenes, dioxins, fluorobenzoate, furfural, and nitrotoluene. Additionally, four orthogroups were annotated as cytochrome P450, which is a broad-substrate-specificity oxygenase capable of catalyzing various detoxification reactions such as hydroxylation, dealkylation, sulfurization, epoxidation, and reduction (Lin et al., 2022).

The majority of genes associated with xenobiotic biodegradation and metabolism pathways in our metagenome were found to be encoded by Pantoea, Sphingomonadales, Pseudomonas, and Methylobacteriaceae. Consistent with previous findings (Chen et al., 2021), taxa such as *Pantoea*, *Sphingomonas*, and *Pseudomonas* were dominant in the tobacco phyllosphere samples exposed to a 50% pesticide concentration. Moreover, studies have demonstrated the abilities of *Sphingomonas* species to degrade various organic pollutants, including phenol (Gong et al., 2016), bisphenol (Fujiwara et al., 2016), phenanthrene (Liu et al., 2016), nicotine, astaxanthin and chlorogenic acid (Ma et al., 2016), triclocarban (Mulla et al., 2016), γ-hexachlorocyclohexane (Tabata et al., 2013), nonylphenol polyethoxylates (Bai et al., 2016), hexachlorocyclohexane isomers (Kumari et al., 2002), plasticizers (Kera et al., 2016), and dioxin (Miller et al., 2010).

These findings highlight the potential of phyllosphere microbes, particularly those belonging to taxa such as *Pantoea* and *Sphingomonas*, to play a crucial role in the degradation of organic pollutants and the maintenance of plant health in polluted environments.

### Quorum sensing

Quorum sensing (QS) is a crucial mechanism of microbial communication in the phyllosphere, allowing for coordinated phenotypic and behavioral responses through diffusible signal molecules, including biofilm formation, virulence, and pathogenicity (Lv et al., 2012). In our metagenome analysis, we identified a significant number of genes (381 gene orthogroups) associated with quorum sensing (**Figure 4B** and see Table S1 at https://doi.org/10.6084/m9.figshare.21257352.v1). These genes include N-acyl homoserine lactone hydrolase, acyl-homoserine lactone synthase, acyl-homoserine-lactone acylase, diguanylate cyclase, LsrR operon transcriptional repressor, and LuxR family transcriptional regulator that bind to homoserine lactones and activate respective operon genes. These genes are predominantly found in Gammaproteobacteria such as *Enterobacter*, *Escherichia*, as well as Alphaproteobacteria including Rhizobiaceae, Methylobacteriaceae, and Sphingomonadales. Specifically, acyl-homoserine lactone synthases are mainly encoded by Methylobacteriaceae and *Pantoea*, while diguanylate cyclases are primarily encoded by *Enterobacter* and *Pantoea*. Diguanylate cyclases are responsible for catalyzing the synthesis of cyclic di-GMP, a critical signaling molecule in quorum sensing, which is known to regulate biofilm formation and decrease motility (Antoniani et al., 2010). The predominant presence of diguanylate cyclase genes in *Enterobacter* and *Pantoea* aligns with previous studies highlighting their involvement in these processes (Bible et al., 2021; Wang et al., 2021).

The identification of genes related to quorum sensing in the tobacco phyllosphere microbiome suggests its potential importance in regulating various microbial behaviors and phenotypes. These findings provide valuable insights into the communication and coordination among phyllosphere microorganisms.

### Oxidative stress resistance and sulfur metabolism

Oxidative stress occurs as a result of the excessive production of reactive oxygen species (ROS), which can cause damage to various cellular biomolecules and disrupt redox regulation. Various abiotic stresses such as drought (Niu et al., 2021), osmotic pressure (Cai-Hong et al., 2005), toxic metal stress (Schützendübel and Polle, 2002) contribute to the development of oxidative stress. However, the introduction of microbes to plants, whether naturally present or through exogenous inoculation, can enhance tolerance to oxidative stress by inducing the synthesis and secretion of antioxidants (Ilyas et al., 2021). Plant growth-promoting bacteria (PGPB) are particularly important in mitigating oxidative stress through the utilization of microbial antioxidant enzymes, which effectively scavenge ROS and maintain a balance between ROS production and removal mechanisms through plant-microbial interactions (Shaffique et al., 2022).

**Figure 5:**
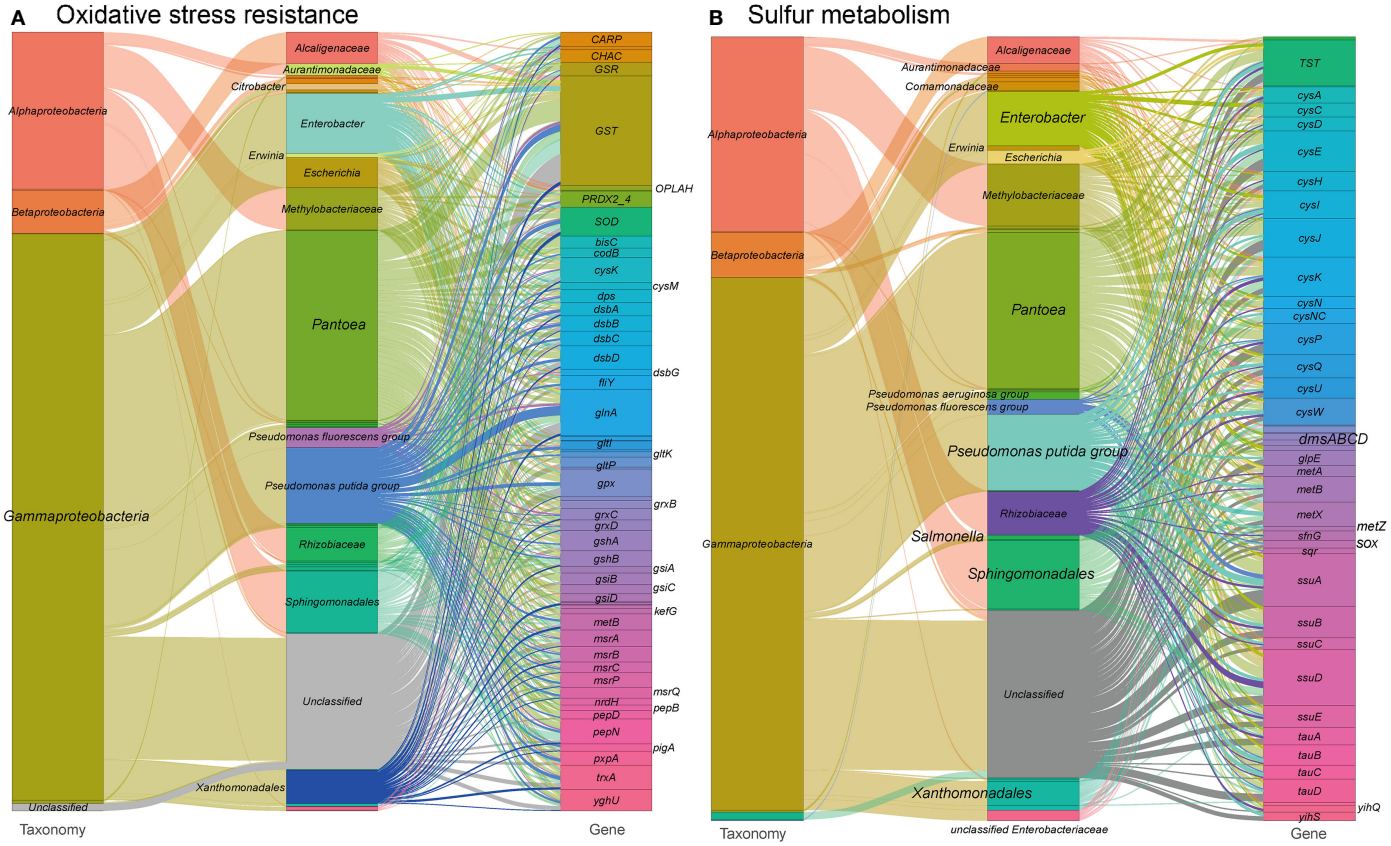
Sankey diagram depicting the taxonomic and functional profiles of genes conferring abiotic stress resilience in the metagenome of cigar tobacco phyllospheric microbiome: **(A)** Oxidative stress resistance; **(B)** Sulfur metabolism. Details for gene abbreviations can be found in Table S1 at https://doi.org/10.6084/m9.figshare.21257352.v1.

As illustrated in our findings, the phyllosphere microorganisms in tobacco harbor a substantial number of genes related to the canonical antioxidant systems (Gill and Tuteja, 2010; Hasanuzzaman et al., 2019) (see **Figure 5A** and Table S1 at https://doi.org/10.6084/m9.figshare.21257352.v1). These genes encode proteins involved in thiol:disulfide interchange, antioxidant enzymes, amino acid transporters, glutathione biosynthesis, glutaredoxins, ion transporters, and other components of the antioxidant defense system. Gammaproteobacteria, including *Enterobacter, Escherichia, Pseudomonas*, and Alphaproteobacteria, including Rhizobiaceae, Methylobacteriaceae, and Sphingomonadales, are the major contributors of these genes. Notably, *Pseudomonas* encodes a significant proportion of heme oxygenase and Fe-Mn superoxide dismutase (SOD), which are crucial components of the antioxidant defense system. We also detected genes encoding components of the oxidative electron transfer chain, such as cytochrome oxidase, that are likely involved in oxidative stress and acid stress tolerance (de la Garza-García et al., 2021). Additionally, we found genes encoding isocitrate dehydrogenase, which may contribute to a supply of reductant NADPH for defending against oxidative stress (Komatsu et al., 2014).

Sulfur metabolism (**Figure 5B** and see Table S1 at https://doi.org/10.6084/m9.figshare.21257352.v1) is closely related to the biosynthesis of antioxidant molecules, such as cystathionine. Genes involved in the inorganic sulfur metabolism pathway, including the *cysACDEHIJKNPQUW* operon and the bifunctional enzyme *cysNC*, are mainly encoded by *Enterobacter* in the metagenome. Other sulfur metabolism genes encode enzymes and transporters related to sulfur oxidation, cystathionine synthesis, taurine metabolism, sulfide oxidation, thiosulfate metabolism, and organic sulfur metabolism. Many of these genes are predominantly encoded by Sphingomonadales, *Pseudomonas*, and Rhizobiaceae, with specific functions such as the metabolism of sulfoquinovose and sulfoquinovosidase (Sharma et al., 2021). The sulfur metabolism pathways in the metagenome are mainly encoded by *Pantoea, Pseudomonas*, and Methylobacteriaceae.

Overall, our findings suggest that the phyllosphere microbiome in tobacco possesses a diverse array of genes involved in antioxidant systems and sulfur metabolism, which contribute to oxidative stress protection and the modulation of redox regulation. These microbial functions are likely to play a crucial role in enhancing the plant’s ability to cope with oxidative stress.

### Acid tolerance and nitrogen metabolism

**Figure 6:**
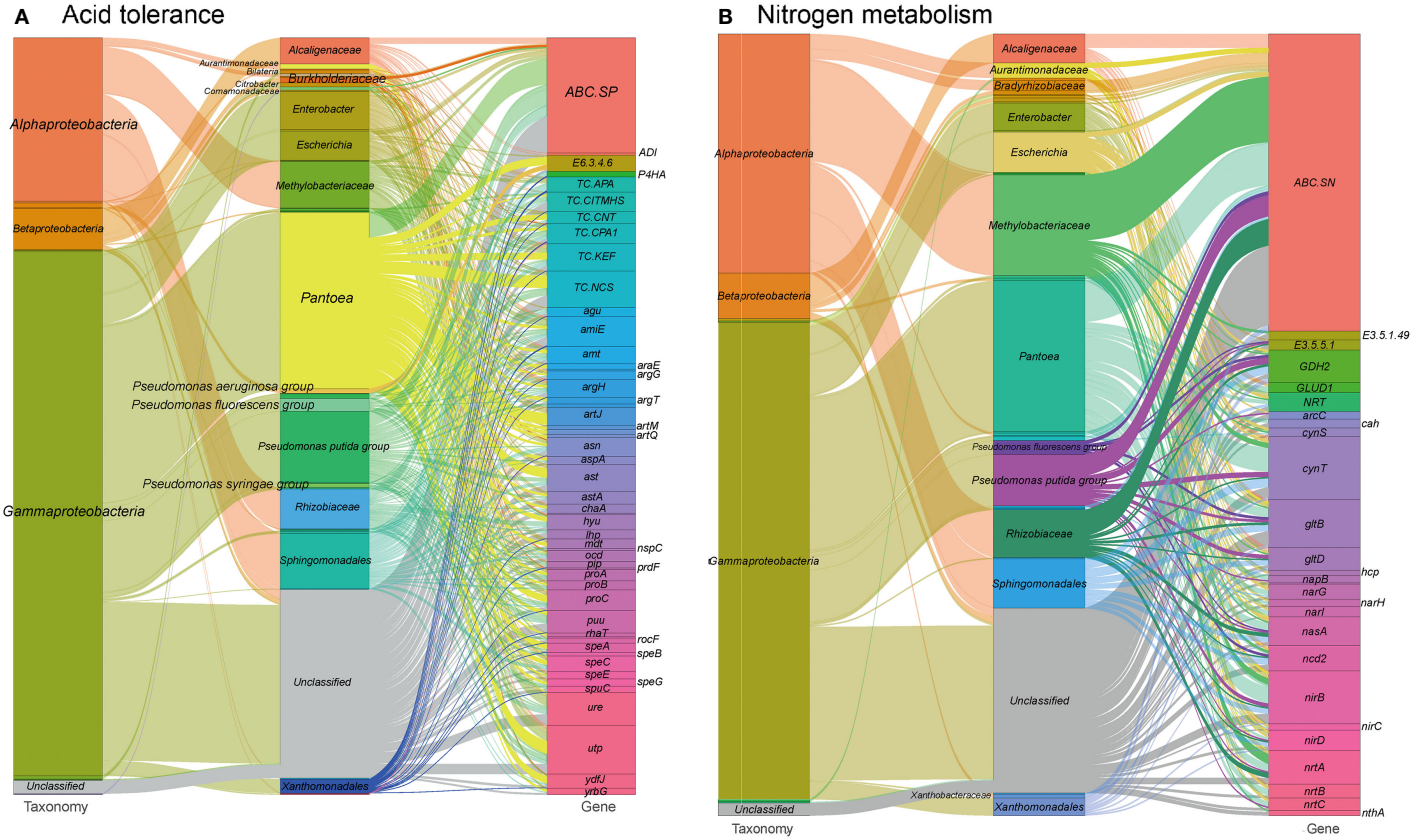
Sankey diagram that illustrates the taxonomic and functional profiles of genes conferring abiotic stress resilience in the cigar tobacco phyllospheric microbiome: **(A)** Acid resistance; **(B)** Nitrogen metabolism. Details for gene abbreviations can be found in Table S1 at https://doi.org/10.6084/m9.figshare.21257352.v1.

Environmental pH perturbation is a significant factor affecting both plants and their associated microbiota, as it can disrupt cellular homeostasis and physiology (Zhou et al., 2022). The southwestern regions of China have experienced high levels of acid rain, which may have had an impact on foliar ecology (Zhang et al., 2021). In our study, we identified 1,373 gene entries related to acid stress resistance in the tested metagenomes (see Table S1 at https://doi.org/10.6084/m9.figshare.21257352.v1). These genes are mainly encoded by *Pantoea* (22.0%), *Pseudomonas* (12.6%), *Sphingomonadales* (6.9%) and *Methylobacteriaceae* (6.0%) (**Figure 6A**). These genes encode proteins involved in acid stress chaperoning, transport systems for basic amino acids, spermidine, putrescine, urea, ammonium, and protons, as well as enzymes for arginine metabolism and urease production. These proteins play roles in pH regulation by neutralizing protons with basic products like arginine and polyamines (Stincone et al., 2011). Additionally, the ion/proton transporters related to osmotic pressure tolerance mentioned earlier could also contribute to adaptive responses to pH perturbation (Liang et al., 2020).

Furthermore, we identified 622 gene orthogroups involved in nitrogen metabolism (**Figure 6B** and see Table S1 at https://doi.org/10.6084/m9.figshare.21257352.v1). These gene orthogroups include genes encoding transporters (ABC.SN/NitT), enzymes (formamidase, nitrilase etc.), and other nitrogen metabolism-related proteins. These genes might also play a role in modulating pH homeostasis through the production of basic substrates, such as ammonium, and promoting nitrogen source utilization in the phyllosphere microbiome and the plant host. Nitrogen metabolism pathways in the metagenomes are primarily encoded by *Pantoea* (19.3%), *Pseudomonas* (9.0%) and *Methylobacteriaceae* (12.8%). Specially, glutamate dehydrogenase (GDH2) is mainly encoded by *Sphingomonadales* (26.0%), and *Pseudomonas* (26.0%), whereas nitronate monooxygenase (*ncd2*) is mainly encoded by *Sphingomonadales* (20.0%) and ferredoxin-nitrite reductase (*nirA*) is mainly encoded by *Methylobacteriaceae* (75.0%).

These findings suggest that the phyllosphere microbiome possesses genetic resources for adapting to acid stress and modulating pH homeostasis through mechanisms such as proton neutralization and nitrogen metabolism. However, further investigations are needed to understand the specific roles of these genes and their interactions in the context of environmental pH perturbation.

### Ultraviolet light damage resistance

DNA can undergo damage from various abiotic factors, such as ultraviolet (UV) light radiation resulting from prolonged sun exposure. This exposure can induce oxidative damage and cross-links between DNA and proteins or DNA strands. The accumulation of such damages can ultimately lead to genomic instability and cell death (Tuteja et al., 2009).

**Figure 7:**
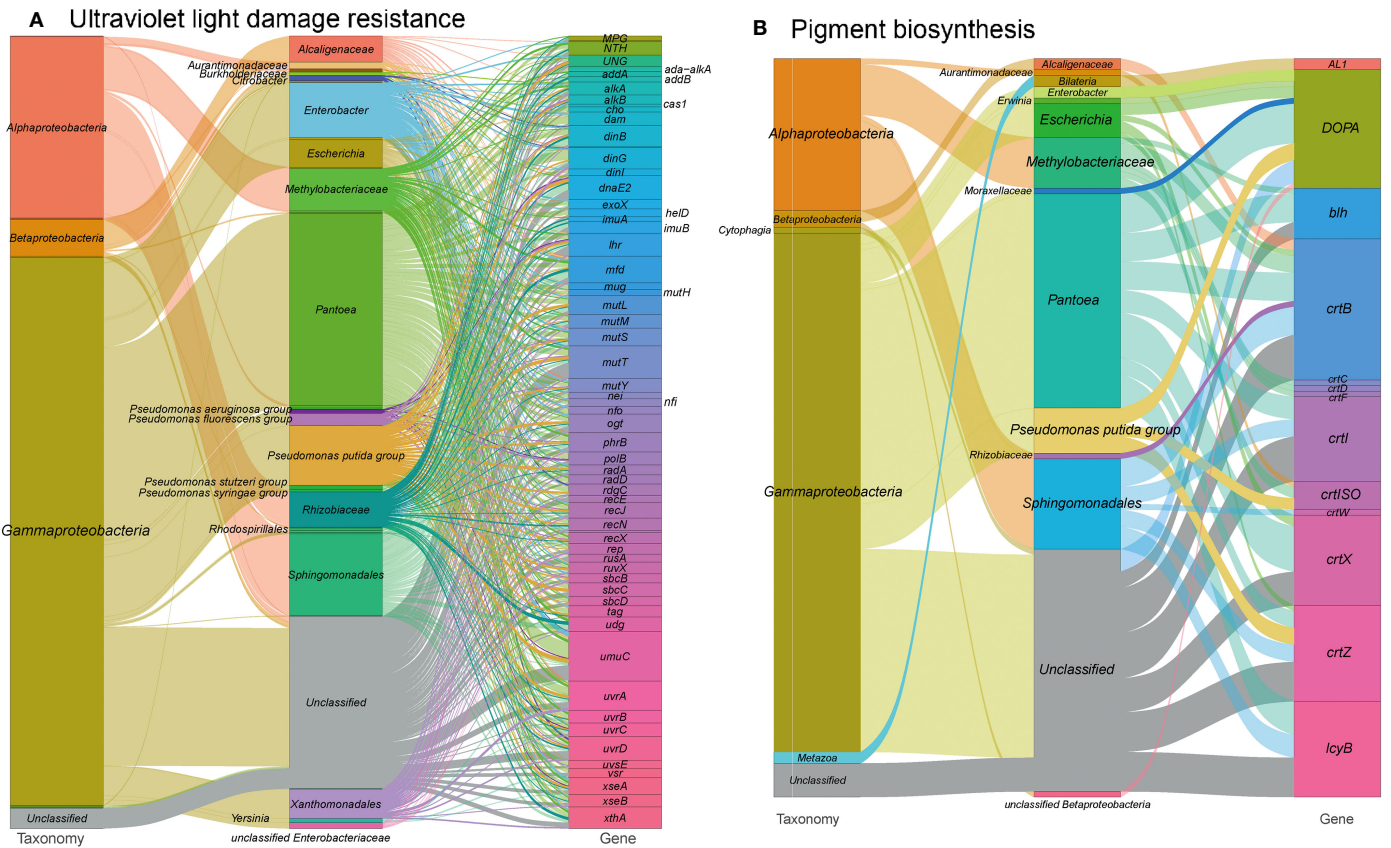
Sankey diagram that illustrates the taxonomic and functional profiles of genes conferring abiotic stress resilience in the cigar tobacco phyllospheric microbiome: **(A)** Ultraviolet light damage resistance; **(B)** Pigment biosynthesis. Details for gene abbreviations can be found in Table S1 at https://doi.org/10.6084/m9.figshare.21257352.v1.

In our analysis of the functional profile of the tobacco microbiome, we observed a complete set of DNA repair machinery, consisting of 1,210 gene orthogroups (**Figure 7A** and see Table S1 at https://doi.org/10.6084/m9.figshare.21257352.v1). These genes are essential for protecting DNA from disruption caused by harmful radiation. They encode proteins involved in nucleotide excision repair (*mfd, uvrABCD*), base excision repair (*ada-alkA, alkA, xthA etc.*), DNA repair and recombination (*addAB, alkB, cas1, etc.*), and finally, mismatch repair (*dam, exoX, mutH, etc.*). Overall, the DNA repair metabolism pathways in our metagenome are mainly encoded by *Pantoea* (22.0%), *Pseudomonas* (12.6%), *Sphingomonadales* (6.9%) and *Methylobacteriaceae* (6.0%). This supports the notion that these microorganisms play a crucial role in protecting DNA from damage caused by harmful radiation. These findings are consistent with our previous study, which showed the enrichment of *Pseudomonas* (LDA = 5.29) and *Sphingomonas* (LDA = 4.19) during T3, a time period characterized by severe sun exposure (Wang et al., 2022).

Furthermore, we identified 132 gene orthogroups related with the production of pigments that absorb radiation (**Figure 7B** and see Table S1 at https://doi.org/10.6084/m9.figshare.21257352.v1), such as carotenoid biosynthesis (AL1, *crtBCDFIWXZ, crtISO, etc.*) and betalain biosynthesis (DOPA). Carotenoid biosynthesis is also linked to the production of phytohormones, such as gibberellins and abscisic acid, which help plants cope with abiotic stresses (Qin et al., 2007; Alagoz et al., 2018). Additionally, we annotated the microbial enzyme aminocyclopropane-1-carboxylic acid (ACC) deaminase, which reduces ethylene levels in host plants and contributes to plant tolerance against abiotic stresses (Ali et al., 2014; Glick and Nascimento, 2021).

Moreover, we found 33 gene orthogroups encoding sporulation proteins (*spoVR, spoVFA, spoIVCA*) and spore photoproduct lyase (*spmBE*), which are involved in abiotic stress tolerance and repair of UV-induced DNA damage in germinating bacterial spores (Table S1 at https://doi.org/10.6084/m9.figshare.21257352.v1) (Nicholson et al., 1997). Methylobacteriaceae, a family of Alpharoteobacteria, predominantly encoded these proteins, potentially explaining their abundance on the plant’s phyllosphere and their tolerance to harmful UV irradiation (Yoshida et al., 2017).

In conclusion, the presence of DNA repair machinery, pigment-producing pathways, and stress-tolerance proteins in the tobacco phyllosphere microbiome suggests a microbial contribution to the plants’ defense against abiotic stresses, including UV radiation. However, further research is needed to establish the specific mechanisms and interactions underlying these processes.

### Viral sequence mining from metagenome

Viruses have been found to play a role in shaping bacterial communities. However, limited research has been conducted on the diversity and abundance of phyllosphere viral communities and their interactions with other microorganisms (Morella et al., 2018). Recently, there has been a growing focus on mining virome information using culture-independent metagenome or metatranscriptome technologies (Forero-Junco et al., 2022; Lauber and Seitz, 2022; Lee and Jeong, 2022). Understanding the virome in plant-associated environments can provide valuable insights into their potential influence on microbial adaptation to various stresses (Xu P. et al., 2008).

**Figure 8:**
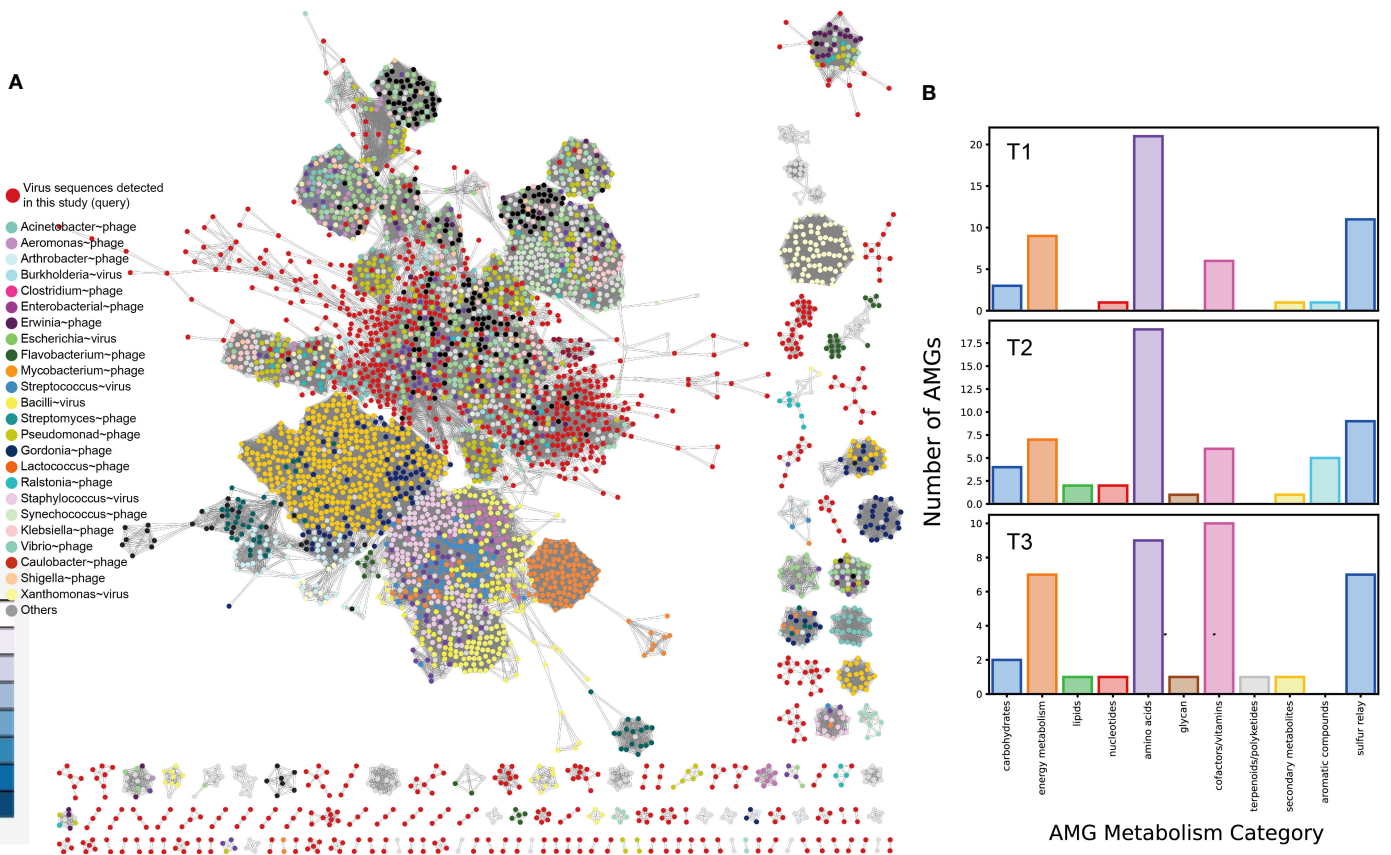
Clustering of detected viral genomes from cigar tobacco phyllosphere metagenome with reference viral genomes based on shared genes. **(A)** vConTACT2 output network. The network was visualized using Cytoscape v3.8.0 (https://cytoscape.org). Queried viruses are colored in red, and reference viruses are color coded based on the host they were annotated to infect; **(B)** Relative abundance of functional categories of identified viral genes per sample.

In this study, the researchers examined a total of 3,320 putative viral scaffolds from the metagenome of the cigar tobacco phyllosphere, divided into T1 (1,141), T2 (1,418), and T3 (761) groups, indicating that the viromes are diverse and dynamic. Out of these examined scaffolds, 2,616 were predicted to be confidential viral genomes (Table S2A at https://doi.org/10.6084/m9.figshare.21257382.v1). The average length of the predicted viral genomes was 2.45 kbp, with the longest being 76,944 bp and the smallest being 1,000 bp. The viral scaffolds contained an average of 4.18 ± 3.40 genes. The quality of the viral genomes varied, with only 0.2% classified as “complete,” 0.3% as “high quality,” 0.5% as “medium quality,” 43.7% as “low quality,” and 55.0% as “not determined” according to CheckV (Nayfach et al., 2021). Out of the predicted viral genomes, 36 were found to be lysogenic based on the presence of proviral sequences. However, it is important to note that bioinformatics prediction often underestimates the number of lysogenic viruses and fails to distinguish between pseudolysogens and lytic viruses (Roux et al., 2015). The tobacco phyllosphere viral genomes were clustered using vConTACT2 (Bin Jang et al., 2019) along with 2,616 known prokaryotic viruses (**Figure 3A**). The tobacco phyllosphere viral genomes were found to be closely related to phages that infect *Pseudomonas* (15.0%), *Burkholderia* (6.4%), *Enterobacteria* (8.4%), *Salmonella* (15.9%), *Escherichia* (14.8%), *Klebsiella* (9.9%), and *Ralstonia* (3.1%) at a taxonomic level higher than genus. It is worth noting that many of these taxa were found to be dominant lineages inhabiting the tobacco phyllosphere such as *Pseudomonas*, as mentioned earlier, and *Ralstonia* spp. is known as a significant tobacco pathogen causing severe bacterial wilt disease (Tao et al., 2022). Furthermore, the sampling site of this study is consistently affected by wildfire disease. It is known that viruses infecting the same type of host often exchange genes or DNA fragments, leading to the formation of strong genotypic clusters (Szymczak et al., 2019). Among the remaining viral populations, 1,400 nodes were classified as unclassified viruses that shared minimal genes with both the database and each other. This highlights the unexplored diversity of the tobacco phyllosphere virome (**Figure 8A**).

Upon closer examination of the genes present in the tobacco phyllosphere virome, a fascinating virus-host interaction is revealed. This interaction seems to maintain a delicate balance between viral predation and host stress resilience, which contributes to the sustainability of the phyllosphere ecosystem. The virome in the tobacco phyllosphere contains a diverse range of genes that potentially confer metabolic functions and resistance to abiotic stresses in the environment.

Through the analysis of the 3,320 viral scaffolds, a total of 15,908 proteins were identified and annotated, as shown in Table S2B at https://doi.org/10.6084/m9.figshare.21257382.v1. Approximately 53% of these proteins were assigned to sequences in the queried databases (KEGG/VOG/Pfam), while around 46.4% were categorized as “uncharacterized” or “hypothetical” proteins. Among the identified proteins, the most prevalent categories were cofactor and vitamin metabolism, amino acid metabolism, energy metabolism, and the sulfur relay system (**Figure 8B**). Notably, many genes within the viral populations appear to be involved in abiotic stress resistance (**Table 1**).

**Table 1:** Auxiliary metabolic genes (AMGs) identified and annotated in viral scaffolds.

AMG KO	AMG count	AMG KO name	KEGG category
T1			
K01186	1	NEU1; sialidase-1 [EC:3.2.1.18]	00600 Sphingolipid metabolism
K01737	1	queD, ptpS, PTS; 6-pyruvoyltetrahydropterin/6-carboxytetrahydropterin synthase [EC:4.2.3.12 4.1.2.50]	00790 Folate biosynthesis
K00558	15	DNMT1, dcm; DNA (cytosine-5)-methyltransferase 1 [EC:2.1.1.37]	00270 Cysteine and methionine metabolism
K01179	1	E3.2.1.4; endoglucanase [EC:3.2.1.4]	00500 Starch and sucrose metabolism
K00390	6	cysH; phosphoadenosine phosphosulfate reductase [EC:1.8.4.8 1.8.4.10]	00920 Sulfur metabolism
K00147	1	proA; glutamate-5-semialdehyde dehydrogenase [EC:1.2.1.41]	00330 Arginine and proline metabolism
K01784	1	galE, GALE; UDP-glucose 4-epimerase [EC:5.1.3.2]	00052 Galactose metabolism
K10026	1	queE; 7-carboxy-7-deazaguanine synthase [EC:4.3.99.3]	00790 Folate biosynthesis
K21140	9	mec; [CysO sulfur-carrier protein]-S-L-cysteine hydrolase [EC:3.13.1.6]	04122 Sulfur relay system
K00121	1	frmA, ADH5, adhC; S-(hydroxymethyl)glutathione dehydrogenase/alcohol dehydrogenase [EC:1.1.1.284 1.1.1.1]	00010 Glycolysis/Gluconeogenesis
K01495	1	GCH1, folE; GTP cyclohydrolase IA [EC:3.5.4.16]	00790 Folate biosynthesis
K01939	1	purA, ADSS; adenylosuccinate synthase [EC:6.3.4.4]	00230 Purine metabolism
K01607	1	pcaC; 4-carboxymuconolactone decarboxylase [EC:4.1.1.44]	00362 Benzoate degradation
K16066	1	ydfG; 3-hydroxy acid dehydrogenase/malonic semialdehyde reductase [EC:1.1.1.381 1.1.1.-]	00240 Pyrimidine metabolism
K06920	2	queC; 7-cyano-7-deazaguanine synthase [EC:6.3.4.20]	00790 Folate biosynthesis
T2			
K00558	17	DNMT1, dcm; DNA (cytosine-5)-methyltransferase 1 [EC:2.1.1.37]	00270 Cysteine and methionine metabolism
K00147	1	proA; glutamate-5-semialdehyde dehydrogenase [EC:1.2.1.41]	00330 Arginine and proline metabolism
K00390	7	cysH; phosphoadenosine phosphosulfate reductase [EC:1.8.4.8 1.8.4.10]	00920 Sulfur metabolism
K01179	1	E3.2.1.4; endoglucanase [EC:3.2.1.4]	00500 Starch and sucrose metabolism
K00287	1	DHFR, folA; dihydrofolate reductase [EC:1.5.1.3]	00790 Folate biosynthesis
K01737	1	queD, ptpS, PTS; 6-pyruvoyltetrahydropterin/6-carboxytetrahydropterin synthase [EC:4.2.3.12 4.1.2.50]	00790 Folate biosynthesis
K16066	1	ydfG; 3-hydroxy acid dehydrogenase/malonic semialdehyde reductase [EC:1.1.1.381 1.1.1.-]	00240 Pyrimidine metabolism
K21140	11	mec; [CysO sulfur-carrier protein]-S-L-cysteine hydrolase [EC:3.13.1.6]	04122 Sulfur relay system
K01607	1	pcaC; 4-carboxymuconolactone decarboxylase [EC:4.1.1.44]	00362 Benzoate degradation
K15634	2	gpmB; probable phosphoglycerate mutase [EC:5.4.2.12]	00010 Glycolysis/Gluconeogenesis
K06920	1	queC; 7-cyano-7-deazaguanine synthase [EC:6.3.4.20]	00790 Folate biosynthesis
K10026	1	queE; 7-carboxy-7-deazaguanine synthase [EC:4.3.99.3]	00790 Folate biosynthesis
K01495	1	GCH1, folE; GTP cyclohydrolase IA [EC:3.5.4.16]	00790 Folate biosynthesis
T3			
K21140	7	mec; [CysO sulfur-carrier protein]-S-L-cysteine hydrolase [EC:3.13.1.6]	04122 Sulfur relay system
K00558	8	DNMT1, dcm; DNA (cytosine-5)-methyltransferase 1 [EC:2.1.1.37]	00270 Cysteine and methionine metabolism
K01626	1	E2.5.1.54, aroF, aroG, aroH; 3-deoxy-7-phosphoheptulonate synthase [EC:2.5.1.54]	00400 Phenylalanine, tyrosine and tryptophan biosynthesis
K00059	1	fabG, OAR1; 3-oxoacyl-[acyl-carrier protein] reductase [EC:1.1.1.100]	00061 Fatty acid biosynthesis
K00737	1	MGAT3; beta-1,4-mannosyl-glycoprotein beta-1,4-N-acetylglucosaminyltransferase [EC:2.4.1.144]	00510 N-Glycan biosynthesis
K08289	1	purT; phosphoribosylglycinamide formyltransferase 2 [EC:2.1.2.2]	00230 Purine metabolism
K13315	1	eryBII, tylCII, tylC1, calS12, atmS12; NDP-hexose C3-ketoreductase/dTDP-4-oxo-2-deoxy-alpha-D-pentos-2-ene 2,3-reductase [EC:1.1.1.-]	00523 Polyketide sugar unit biosynthesis
K01737	1	queD, ptpS, PTS; 6-pyruvoyltetrahydropterin/6-carboxytetrahydropterin synthase [EC:4.2.3.12 4.1.2.50]	00790 Folate biosynthesis
K06211	1	nadR; HTH-type transcriptional regulator, transcriptional repressor of NAD biosynthesis genes [EC:2.7.7.1 2.7.1.22]	00760 Nicotinate and nicotinamide metabolism
K01113	1	phoD; alkaline phosphatase D [EC:3.1.3.1]	00790 Folate biosynthesis
K10026	1	queE; 7-carboxy-7-deazaguanine synthase [EC:4.3.99.3]	00790 Folate biosynthesis
K00390	7	cysH; phosphoadenosine phosphosulfate reductase [EC:1.8.4.8 1.8.4.10]	00920 Sulfur metabolism
K00287	1	DHFR, folA; dihydrofolate reductase [EC:1.5.1.3]	00790 Folate biosynthesis
K06920	1	queC; 7-cyano-7-deazaguanine synthase [EC:6.3.4.20]	00790 Folate biosynthesis
K01625	1	eda; 2-dehydro-3-deoxyphosphogluconate aldolase/(4S)-4-hydroxy-2-oxoglutarate aldolase [EC:4.1.2.14 4.1.3.42]	00030 Pentose phosphate pathway
K01495	1	GCH1, folE; GTP cyclohydrolase IA [EC:3.5.4.16]	00790 Folate biosynthesis

For example, the virome assembled from the metagenome of the T3 sample contained an alkaline phosphatase D (*phoD*), which may contribute to the solubilization of inorganic insoluble phosphorus, thereby enhancing environmental phosphorus availability (Thapa et al., 2017). Additionally, a glutamate-5-semialdehyde dehydrogenase (*proA*) was identified, which is involved in the synthesis of the osmolyte proline. Furthermore, numerous viruses carry genes related to cysteine and methionine metabolism, which are closely linked to oxidative stress resistance. These include genes such as phosphoadenosine phosphosulfate reductase (*cysH*, 20 entries), DNA (cytosine-5)-methyltransferase (*dcm*, 40 entries). It is noteworthy that these relatively abundant genes may confer benefits to viral populations, considering their detection in multiple samples, despite the presumably high maintenance cost associated with the presence of such genes in viral genomes. Moreover, the viral sequences also contain genes associated with the metabolism of protectant sugars, such as endoglucanase, NDP-hexose ketoreductase (*eryBII*), UDP-glucose 4-epimerase (*galE*), beta-1,4-N-acetylglucosaminyltransferase (MGAT3), which may contribute to the breakdown of complex polysaccharides abundant on plant surfaces (Xu et al., 2008). Additionally, genes involved in the degradation of organic pollutants, such as 4-carboxymuconolactone decarboxylase (*pcaC*), were also found.

Genes related to stress resilience and auxiliary metabolic functions were identified across all samples, with the highest abundance observed in the T2 group and the highest diversity in the T3 group (**Figure 8A**). It is proposed that these viruses may serve as vectors for horizontal gene transfer (HGT) and deliver stress resilience genes to their host counterparts. The emergence and mechanism of HGT events (“Why does lateral transfer occur in so many species and how?”) is recommended as a still-pending and significant scientific question by the editorial of the journal Science in the article “So much more to know” (American Association for the Advancement of Science, 2005), and viruses could be instrumental in this process. Notably, (pseudo)lysogenic viruses carrying tolerance genes may enhance microbial survival on the plant phyllosphere by taking refuge in the host cytoplasm or genome. This mutualistic interaction aligns with similar proposals in previous studies, such as marine cyanophages carrying photosystem genes (Sullivan et al., 2006) and mangrove soil viruses carrying carbohydrate-active enzymes (Jin et al., 2019). Viruses may also facilitate the formation and dispersal of microbial biofilms, providing them shelter in harsh environments (Rice et al., 2009; McDougald et al., 2011; Secor et al., 2015). Biofilms have even been observed in hot desert soil, where temperate viruses are suggested to be positively selected (Zablocki et al., 2016; Lebre et al., 2017).

The phyllosphere microbiota faces hostile abiotic conditions, such as high UV radiation, which can pose challenges for viruses (Iriarte et al., 2007). These conditions may have led to the selection of phages that are adapted to such pressures. Similarly, in the tobacco phyllosphere, viruses carrying stress resilience genes may improve their survival and reproduction by integrating their DNA into a bacterial host genome and aiding the host in thriving under abiotic stresses. However, it is worth noting that most of the identified viruses in the tobacco phyllosphere were predicted to be lytic, meaning they undergo a lytic cycle when environmental conditions are more favorable, such as during rainfall. The high ratio of lytic to lysogenic phages may be a result of the low microbial densities on the plant phyllosphere, which is consistent with the “kill-the-winner” dynamics, leading to a higher prevalence of lytic phages (Knowles et al., 2016). This finding is consistent with a previous report on the wheat phyllosphere (Forero-Junco et al., 2022). Nonetheless, experimental validation of a host-virus model specific to the tobacco phyllosphere is essential to confirm these hypotheses and determine the extent to which virus-mediated resilience genes contribute to enhancing microbial fitness under abiotic stresses.

## Conclusions

In conclusion, our study utilized shotgun metagenomic sequencing to investigate the functional profile of the phyllosphere microbiota in tobacco plants and identify potential plant growth promoting bacteria (PGPB) that confer abiotic stress resilience. Our findings reveal the importance of microbial associations in mediating plant protection and responses to various stressors.

We observed that abundant genes from bacterial lineages, particularly *Pseudomonas*, within the cigar tobacco phyllospheric microbiome contribute to resilience against osmotic and drought stress, heavy metal toxicity, temperature perturbation, organic pollutants, oxidative stress resistance, and UV light damage. This highlights the crucial role of bacteria in enhancing stress tolerance in the phyllosphere.

Furthermore, our virome mining analysis unveiled the presence of viruses within the phyllosphere microbiome, including phages infecting *Pseudomonas, Burkholderia, Enterobacteria, Ralstonia*, and other related viruses. We identified genes associated with abiotic stress resilience in the virome, such as alkaline phosphatase D (*phoD*) and glutamate-5-semialdehyde dehydrogenase (*proA*), which contribute to nutrient solubilization and osmolyte synthesis, respectively.

These novel findings underscore the unexplored roles of viruses in facilitating and transferring abiotic stress resilience in the phyllospheric microbiome through beneficial virus-host interactions. By expanding our understanding of the taxonomic and functional profiles of abiotic stress resilience in the phyllosphere, this study provides valuable insights for the selection of PGPB candidates from the tobacco phyllosphere to enhance stress tolerance in plants.

Overall, our research enhances our knowledge on the intricate relationships between microorganisms and plants, advancing our understanding of the mechanisms underlying abiotic stress resilience. These findings have important implications for agricultural practices, as they can contribute to the development of strategies to enhance stress tolerance in crop plants by harnessing the potential of the phyllosphere microbiome.

## Materials and methods

### Sample collection

Samples of cigar tobacco (*Nicotiana tabacum* L.) leaves were collected from Yongding County, Zhangjiajie City, Hunan Province, China (29.13° N, 110.48° E) in June (T1), July (T2), and August (T3) of 2021. This sampling region is continuingly affected by bacterial wildfire disease. For each replicate, 5–7 middle leaves from a 90 m^2^ plot area were randomly selected. This area was affected by bacterial wildfire disease. The leaf samples were stored in sterile plastic bags, transported to the laboratory, and stored at 4°C for subsequent foliar microbial DNA extractions. A total of over 120 leaves from thirty cigar tobacco plants were used in this study, representing the three time points (T1, T2, and T3) in three biological duplicates.

### DNA extraction and shotgun metagenomic sequencing

In the DNA extraction and shotgun metagenomic sequencing procedures, leaf samples were collected from various parts of the leaf surface (excluding the main and branch veins) using a sterile puncher. A total of 15 grams of leaf samples were collected. The collected leaf samples were transferred into a 250-mL conical flask containing 200 mL of 0.1% Tween-80 bacterial phosphate buffer at pH 7.0. The flask was shaken for 30 minutes at 170 rpm and 25°C. This shaking step was performed to remove the epiphytic microbes from the leaf surface. After shaking, the bacterial suspension was collected by centrifugation at 10,000 rpm for 15 minutes at 4°C. The sediment obtained from the centrifugation was washed three times with sterile water. Finally, the sediment was resuspended with 1 mL of sterile water for DNA extraction purposes. Genomic DNA extraction was performed using the Plant Genomic DNA Kit following the manufacturer’s protocol. The extracted DNA’s quality was checked using a 1.0% agarose gel, and the DNA concentrations were measured using a NanoDrop 1000 spectrophotometer. To ensure replicability and reliability, three types of samples were prepared corresponding to the three time points (T1, T2, and T3), and each time point had three replicates.

For shotgun metagenomic sequencing, the extracted DNA samples were fragmented using ultrasound into approximately 350 bp fragments. These fragments were used to construct sequencing libraries using the NEBNext^®^ Ultra™ DNA Library Prep Kit for Illumina. The libraries were sequenced using the Illumina NovaSeq 6000 Sequencer. The combined datasets of the three groups of leaf samples contained a total of 54.8 Gbp of raw reads.

### Shotgun metagenomic assembly and annotation

For the shotgun metagenomic data, the raw reads were trimmed with the sliding window approach to generate the QC (Quality Control) reads with Trimmomatic (Bolger et al., 2014). Contamination of reads originating from the host plant was aligned to the nuclear genome of *Nicotiana tabacum* TN90 (GCA_000715135.1) using Bowtie 2 (Langmead and Salzberg, 2012). After that, the concordantly mapped reads were removed to preserve the clean reads. To obtain the microbial reads and their taxonomic annotations, the clean reads were aligned using Kraken 2 (Wood et al., 2019), and the reads that could not be aligned to bacteria, fungi, archaea or virus were filtered out. The microbial reads from all the samples were pooled together for *de novo* assembly with MEGAHIT (Li et al., 2015) (-k-min 21, –k-max 191, –min-contig-len 500). For the assembled contigs, ORF (Open Reading Frame) were predicted with Prodigal (Hyatt et al., 2010) in metagenomics mode (–meta). This was followed by protein sequence clustering and analysis and through software BPGA v.1.0 by default procedures. The size of the pan-metagenome was extrapolated by implementing an power law regression function, P_s_ = κn^γ^, using a built-in program of the BPGA pipeline (Chaudhari et al., 2016), in which P_s_ represents the total number of non-orthologous gene families within its pan-metagenome, n represents the number of tested metagenomes, and both κ and γ are free parameters. An exponent γ of <0 suggests the pan-metagenome is “closed,” where the size of the pan-metagenome reaches a constant value as extra metagenomes are added. Conversely, the species is predicted to harbor an open pan-metagenome for γ values between 0 and 1. In addition, the size of the core genome was extrapolated by fitting into an exponential decay function, F_c_ = κ_c_exp(-n/τ_c_), with a built-in program of the BPGA pipeline (Chaudhari et al., 2016), where F_c_ is the number of core gene families, κ_c_, and τ_c_ are free parameters.

For gene annotation, was used to annotate with eggNOG-mapper v.2.0 (Huerta-Cepas et al., 2019) searching with Diamond method (Buchfink et al., 2015) against multiple databases, including COG database (https://www.ncbi.nlm.nih.gov/research/cog/) and KEGG database (Aramaki et al., 2020) (–evalue 0.001 –score 60 –pident 40 –query_cover 20 –subject_cover 20). The orthogroup clustering and gene ontology (GO) categories were performed using OrthoVenn v.2.0 (Xu et al., 2019), and the hypergeometric test with a *p*-value < 0.05 was applied to find enriched GO in the clusters.

### Prediction and analysis of viral scaffolds from metagenome

VIBRANT v1.2.1 (Kieft et al., 2020) with default settings was used for viral signal prediction across the assembled metagenomes and scaffolds length of ≥1,000 bp. CheckV v0.6.0 (Nayfach et al., 2021) was used for completeness and quality estimation. A viral contig was determined as lytic or lysogenic by VIBRANT v1.2.1 (Kieft et al., 2020) (-virome mode -l 5000). VIBRANT uses HMM profiles from three different databases: Kyoto Encyclopedia of Genes and Genomes (KEGG) KoFam (March 2019 release) (Aramaki et al., 2020), Pfam (v32) (El-Gebali et al., 2019), and Virus Orthologous Groups (VOG) (release 94, http://vogdb.org/). VIBRANT-predicted viral scaffolds with qualities of medium, high, and complete were annotated with KEGG, Pfam, and VOG HMMs (hmmsearch (v3.1), e-value < 1e−5) (Eddy, 1998). To identify viral contigs that were grouped with viral RefSeq genomes across the samples, output from vConTACT2 (v.0.9.8) (Bin Jang et al., 2019) for each sample was merged together to create a condensed network, visualized in Cytoscape v3.8.0 (https://cytoscape.org).

## Data availability statement

The datasets presented in this study can be found in online repositories. The names of the repository/repositories and accession number(s) can be found in the article/supplementary material.

## Author contributions

LL and HY conceived and designed the research. ZW, DP, CF, XL, SG and LL analyzed the data. LL wrote the manuscript. All authors contributed to the article and approved the submitted version.
